# Alzheimer's Disease Risk Assessment Using Large-Scale Machine Learning Methods

**DOI:** 10.1371/journal.pone.0077949

**Published:** 2013-11-08

**Authors:** Ramon Casanova, Fang-Chi Hsu, Kaycee M. Sink, Stephen R. Rapp, Jeff D. Williamson, Susan M. Resnick, Mark A. Espeland, for the Alzheimer's Disease Neuroimaging Initiative

**Affiliations:** Wake Forest School of Medicine, Winston-Salem, North Carolina, United States of America; Nathan Kline Institute and New York University School of Medicine, United States of America

## Abstract

The goal of this work is to introduce new metrics to assess risk of Alzheimer's disease (AD) which we call AD Pattern Similarity (AD-PS) scores. These metrics are the conditional probabilities modeled by large-scale regularized logistic regression. The AD-PS scores derived from structural MRI and cognitive test data were tested across different situations using data from the Alzheimer's Disease Neuroimaging Initiative (ADNI) study. The scores were computed across groups of participants stratified by cognitive status, age and functional status. Cox proportional hazards regression was used to evaluate associations with the distribution of conversion times from mild cognitive impairment to AD. The performances of classifiers developed using data from different types of brain tissue were systematically characterized across cognitive status groups. We also explored the performance of anatomical and cognitive-anatomical composite scores generated by combining the outputs of classifiers developed using different types of data. In addition, we provide the AD-PS scores performance relative to other metrics used in the field including the Spatial Pattern of Abnormalities for Recognition of Early AD (SPARE-AD) index and total hippocampal volume for the variables examined.

## Introduction

The development of biomarkers for early detection of Alzheimer's disease (AD) has become an area of intensive research in neuroimaging and genetics. AD has no known cure and is one of the ten main causes of death in US, making it a leading public health concern and imposing a huge economic burden on individuals and society. It is believed that the neurodegenerative processes that lead to AD start many years before the symptoms appear. Earlier detection of the disease would allow earlier interventions and may provide clues to its causes. The Alzheimer's Disease Neuroimaging Initiative (ADNI-1) project [1] collected laboratory, imaging, clinical, cognitive, and genetic data on a large U.S. cohort between the ages of 55 and 90 for 3 years with the goal of identifying biomarkers for early detection of AD. Analyzing the massive amount of information in neuroimaging and genetic datasets such as ADNI is a challenging enterprise that poses great difficulties to traditional statistical methods [2]. Machine learning techniques are being increasingly used in the context of research for early detection of AD because they are well-suited to deal with high-dimensional data.

In this work we introduce new metrics for assessing AD risk based on structural MRI (sMRI) and cognitive performance data using large-scale machine learning methods. There are several existing indices for AD risk assessment that often are generated by severely reducing the dimensionality of imaging and/or genetic data before applying a classification algorithm such as a support vector machine (SVM). For example, the Spatial Pattern of Abnormalities for Recognition of Early AD (SPARE-AD) index [3], [4] and the Structural Abnormality Index (STAND) score [5] were introduced as sMRI-based metrics to detect AD-like structural patterns that rely on image processing feature selection strategies to provide a SVM with a few dozen, several hundreds or thousands of features for final classification. A new index to assess conversion to AD based on the AD Assessment Scale-Cognitive subscale (ADAS-Cog) and Random Forests (RF) methods [6] was recently proposed [7]. This is a composite score based on a weighted average of ADAS-Cog subscores, which uses measures of variable importance generated by RF as weights. Another composite score based on several cognitive tests available in ADNI was recently introduced by [8] using psychometric theory. An index based on multiple kernel learning (MKL) methods which combines information from different data domains (e.g. MRI, positron emission tomography (PET), and genetic and cognitive data) was recently proposed [9]. MKL is a paradigm for data fusion born in the field of genetics as an extension of SVMs [10]–[12]. Instead of one kernel as in the classical SVM, a weighted sum of kernels is computed where each type of information is encoded by a different kernel and the weights are estimated by solving an optimization problem [13], [14]. Using the MKL decision function, they generate scores called multi-modal disease markers (MMDM).

We propose new metrics for assessment of AD risk based on probabilities as modeled by high-dimensional regularized classifiers. We have recently introduced an approach to automatic classification of brain MRI images in AD that is based on large-scale regularization [15]. Instead of combining dimension reduction with SVM, we use regularized logistic regression (RLR) based on a coordinate-wise descent technique as implemented in the GLMNET library [16], [17]. These classification methods can operate directly in the voxel space using regularization with sparsity properties. In previous work using ADNI data, we compared this approach to a linear SVM voxel-based method proposed by [18] which was one of the top performers when discriminating MRI images of cognitively normal (CN) from AD participants in a recent comparison of MRI data classification methods in the field [19]. By examining intensive computational experiments across different normalization templates, degrees of smoothing, and sample sizes, we observed that regularized logistic regression often performed at a similar or higher level when discriminating CN ADNI participants from participants with AD [15], [20]. This suggested that an index for early detection of AD based on class-conditional probabilities modeled by large scale RLR might be a promising metric for assessment of AD risk. In this study we evaluated the validity of these metrics which we called ‘AD Pattern Similarity’ (AD-PS) scores in different scenarios: (1) Associations with conversion from mild cognitive impairment (MCI) to AD, (2) characterization of cognitive status (CN, MCI and dementia), and (3) detection of effects caused by age and functional status based on the Functional Assessment Questionnaire (FAQ). We also studied the performance of a composite cognitive-anatomical metric that assesses AD risk based on information from both sources. Finally, we provided the performance of the AD-PS scores relative to the SPARE-AD index and the total hippocampal volumes in the different scenarios described above.

## Methods

### ADNI database

The ADNI (adni.loni.ucla.edu) was launched in 2003 by the National Institute on Aging, the National Institute of Biomedical Imaging and Bioengineering, the Food and Drug Administration (FDA), private pharmaceutical companies, and non-profit organizations. Its primary goal was to test whether serial MRI, PET, other biological markers, and clinical and neuropsychological assessment could be combined to measure the progression of MCI and early AD. Determination of sensitive and specific markers of very early AD progression could help researchers and clinicians develop new treatments and monitor their effectiveness, and reduce the time and cost of clinical trials. The Principal Investigator of this initiative is Michael W. Weiner, MD, VA Medical Center and University of California – San Francisco. ADNI recruited from over 50 sites across the U.S. and Canada. The initial goal of ADNI was to recruit 800 adult participants, ages 55 to 90, composed of approximately 200 cognitively normal older individuals to be followed for 3 years, 400 people with MCI to be followed for 3 years, and 200 people with early AD to be followed for 2 years. For up-to-date information about the cohort, see www.adni-info.org.

#### Ethics Statement

We used ADNI-1 subject data collected from 50 clinic sites. Ethics approval was obtained for each institution involved including our Institutional Review Board at Wake Forest Baptist Health. This study was conducted according to Good Clinical Practice guidelines, the Declaration of Helsinki, US 21CFR Part 50– Protection of Human Subjects, and Part 56– Institutional Review Boards, and pursuant to state and federal HIPAA regulations. Study subjects gave written informed consent at enrollment for data collection, sample storage and subsequent use of samples for research, and completed questionnaires approved by each participating site's Institutional Review Board. The data were anonymized before being shared.

### ADNI participants

For the present analysis, we used baseline structural MRI, DNA, and cognitive data from 694 Caucasians. Of those, 188 were CN, 171 had AD, and 335 had MCI at baseline [21]. Among the MCI cases, 153 converted to AD over 3 years of follow-up (cMCI) and 182 remained stable (ncMCI). Cognitive evaluation of 77 ncMCI participants at 36 months or less was missing (censored). Demographic information for the ADNI participants is summarized in Table 1. The list of IDs is provided in the supplementary materials (see Tables S3, S4, S5 and S6 in File S1).

**Table 1:** Demographic data for the 694 ADNI participants, shown across categories of clinical status.

	CN	cMCI	ncMCI	AD
**Number**	188	153	182	171
**Age**	75.9 (5.0)	75.0 (7.0)	75.2 (7.6)	75.5 (7.7)
**Sex (M/F)**	102/86	92/61	136/62	95/76
**Educ. (years)**	16.1	15.7	15.7	14.9
**Hand (R/L)**	173/15	142/11	164/18	159/12
**BMI**	26.4 (4.7)	25.4 (4.7)	26.2 (3.6)	25.6(3.9)
**e4 (Y/N)**	49/139	103/50	84/98	112/59
**GDS**	0.8 (1.13)	1.6 (1.4)	1.5 (1.4)	1.7 (1.4)
**FAQ**	0.1 (0.4)	5.4 (4.7)	2.7 (3.7)	13.1 (6.8)
**MMSE**	29.1 (1.0)	26.6 (1.7)	27.5 (1.8)	23.4 (2.0)

Changes in cognitive status occurred within 36 months of follow-up.

**AD** = Alzheimer's disease; **CN** = cognitively normal; **cMCI** = mild cognitive impairment in subjects who converted to AD; **ncMCI** = MCI subjects who remained stable; **BMI**- Body mass index; **GDS** – Geriatric Depression Scale; **FAQ** = Functional Assessment Questionnaire; **MMSE** = Mini-Mental State Exam.

### Structural MRI data

We used baseline 1.5T T1-weighted MRI data as described in the ADNI acquisition protocol [22]. The ADNI protocol acquires 2 sets of structural data at each visit that are rated for image quality and artifacts by ADNI investigators [22]. To enhance standardization across sites and platforms, the best quality data set then undergoes additional pre-processing, including corrections for gradient non-linearity [23] and intensity non-uniformity [24]. In the present study, these optimally pre-processed images were downloaded from the ADNI database and used for subsequent analyses.

The images were segmented and normalized using the Statistical Parametric Mapping (SPM) software package. Segmentation of the original images into grey matter (GM), white matter (WM), and cerebrospinal fluid (CSF) was performed using the NewSegment tool. Normalization was carried out using Diffeomorphic Anatomical Registration using the Exponentiated Lie algebra (DARTEL) method [25]. First, a study-customized template was generated including the 694 images using the default parameters; then GM, WM, and CSF images were warped to the template, modulated, and smoothed using an isotropic Gaussian kernel of 4 mm. The final resolution of the images was 1.5 mm isotropic. The GM, WM and CSF images of all participants were thresholded by using masks generated from the respective GM, WM and CSF study-customized templates (threshold = 0.5). Each mask contained 205245, 136967 and 66636 voxels respectively. The intersection of these masks was empty in all cases. The images were then vectorized and stored in three matrices of predictors where each row contained the imaging information from one participant and each column contained the information corresponding to one voxel. The SPARE-AD scores for the ADNI participants described in Table 1 were provided by Dr. Davatzikos whose staff posted the SPARE-AD indexes on the ADNI website.

In addition, we used as a classical control measure in our analyses, total hippocampal volume (THV). The data were available in the ADNI website and generated using the software for automated segmentation and parcellation FreeSurfer (FS) V4 [26], [27]. FS automatically labels cortical and subcortical tissue classes using an atlas-based Bayesian segmentation procedure which extracts target regions volumes and cortical thickness, as well as to total intracranial volume (ICV). Extracted Free-Surfer values for two independently processed MP-RAGE images of the same participant were averaged to create a mean value for volumetric and cortical thickness measures for all target regions.

### Cognitive and Functional Data

We used 25 cognitive scores from four memory tests (see Table 2) available in ADNI at baseline that have been used in previous work [7], [8]: the AD Assessment Cognitive Scale (ADAS-Cog), Rey Auditory Verbal Learning test (RAVLT), Logical Memory test and Mini-Mental State Examination (MMSE). These cognitive parameters were selected because a prominent feature of AD is memory impairment. In addition, the ADAS-Cog and MMSE are tests of global cognitive function, and they cover several domains other than memory. The 25 scores were concatenated into vectors that were used as input samples to our classification methods. Information on function came from the FAQ, a proxy-reported assessment of everyday functional abilities associated with cognition. Previous ADNI research has studied its relationships with longitudinal measures of glucose metabolism obtained from PET data [28].

**Table 2:** ADNI cognitive instruments used in this study.

Cognitive Tests and Scores	
**ADAS-Cog**	**RAVLT**
Q1 - Word recall	Trial 1
Q2 - Commands	Trial 2
Q3 - Construction	Trial 3
Q4 - Delayed word recall	Trial 4
Q5 - Naming	Trail 5
Q6 - Ideational praxis	Interference trial
Q7 - Orientation	Immediate recall
Q8 – Word recognition	30 minutes delay
Q9 – Recall instruction	recognition
Q10 – Spoken language	**Logical Memory**
Q11 – Word finding	Immediate
Q12 - Comprehension	Delay
Q14 – Number cancellation	**MMSE**
	Total

### Machine learning methodology

In previous work we proposed the use of regularized logistic regression (RLR) with elastic net regularization for high-dimensional classification of AD sMRI images [15], [20]. The RLR method used here is based on the implementation provided by the GLMNET library [16], which uses a very efficient optimization technique called coordinate-wise descent technique [17]. The general form of the optimization problem solved by the library is of the form:
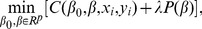
(1)

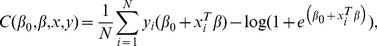
(2)

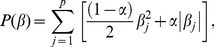
(3)where 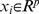 is the i^th^ sample or feature vector containing the i^th^ participant cognitive or MRI data, 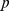 is the number of variables (voxels or cognitive scores) entering the analysis, 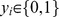 is the i^th^ label (0 for cognitively normal participants, 1 for participants with Alzheimer's disease), 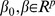 are the parameters of the model, and 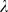 is the regularization parameter. The regularization scheme described by Eq.(1) contains two terms: a loss term 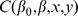 and a penalty term 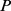 called elastic net penalty, which is given by Eq.(3). The regularization parameter 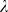 establishes a trade-off between the two terms and it is determined from the data using cross-validation combined with grid search. Our software implementation is based on MATLAB, where the GLMNET library is called using a freely available MATLAB wrapper developed by Hui Jiang (http://www-stat.stanford.edu/~tibs/glmnet-matlab/). Most computations were made on a LINUX computer with 16 CPUs and 96 GBs of RAM. We actively used parallel computing features provided by the MATLAB parallel computing toolbox. The AD-PS scores can be computed overnight using 15 CPUs.

### Optimization of regularization parameters

To estimate the optimal values of the regularization parameters, we combined a three-way split of the data (training-validation-testing) with 10-fold cross-validations (CV) and grid search (see Figure S1 in File S1). This was done to avoid upward bias in the metrics of performance estimates [15], [29], [30]. We implemented an external K_1_-fold CV where at each step we leave one fold for testing and use the remaining K_1_-1 folds for training and validation. These last two procedures are implemented by using a nested K_2_-fold CV. We divide the K_1_-1 folds into K_2_ folds and we leave one fold for validation and K_2_-1 folds for training combined with a grid search to determine the optimal parameters. The grid we used in our analyses was 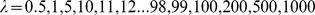. For the sMRI data, we fixed in advance one of the regularization parameters (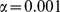) and optimized the second. We have observed in practice working with high-dimensional imaging data that this choice works well avoiding the heavier computational burden related to the optimization of both parameters [20]. At each grid point, the classifier is trained and its performance is assessed using the fold left for validation by estimating the classification accuracy. We select the regularization parameters that produce maximum average accuracy across the K_2_ folds of the internal CV procedure. The classifier is then retrained using the data in the K_1_-1 folds left for training and validation and the selected optimal regularization parameters. The classifier's generalization capability is then evaluated by computing the classification accuracy, sensitivity and specificity using the fold originally left for testing in the external CV. This is repeated K_1_ times and the average classification accuracy is reported. For cognitive data the procedure was similar, but since the problem size is small, we optimized both parameters using a two-dimensional grid (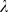 same as above and 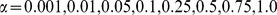). For each type of data, the models were estimated 100 times to account for variability due to random CV partitioning. In our analyses we used K_1_ = 10 and K_2_ = 10.

### Estimation of the AD-PS scores and discriminative maps

The estimation of the AD-PS scores and discriminative maps is based on the CN and AD available in this study's data. The cognitive, GM, WM and CSF AD-PS scores for CN and AD participants are estimated in the external loop of the CV procedure described above to avoid overfitting. The scores for MCI participants were estimated by providing the corresponding data to the classifiers trained with all the available AD and CN data. For each type of data the weights 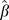 estimated after solving the optimization problem defined by Eqs. (1–3) are replaced in the classical logistic regression model formula for conditional probabilities. The AD-PS score for a given individual will be:
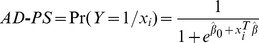
where 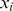 is the structural MRI or cognitive data of the i-th participant. The probability 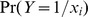 is computed (in practice we take the values returned by the GLMNET software) and the median values of the 100 repetitions were taken as the final values of the scores. Our AD-PS scores are measures of similarity of the biological and clinical patterns (e.g. spatial brain tissue atrophy, cognitive function, etc.) found in a given individual to those found in AD patients.

Finally, voxel-based discriminative maps are generated. The vector 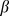 of parameters described in Eqs. (1–3) are estimated using the whole data set and the optimized values of the regularization parameters. These parameters or weights (one per voxel) are then employed to generate the discriminative maps which reflect the brain areas that were more informative when discriminating between the two groups of subjects. The discriminative maps shown later represent the ratio of the average of the weights and their standard deviations obtained across 100 repetitions of the computations to account for variability due to CV partitioning. The areas represented in blue correspond to the negative parameters indicating brain regions associated with AD classification, while the red ones indicate brain areas associated with CN classification.

### Machine learning and statistical analyses

First, we evaluated classifiers' performance across different cognitive groups: 1) CN versus AD; 2) CN versus cMCI; 3) CN versus ncMCI; and 4) ncMCI versus cMCI. In each case RLR models were estimated independently for GM, WM, CSF and cognitive data using in each case all the available samples. Classification accuracy, sensitivity and specificity were estimated based on the nested CV method described above. To account for variability due to CV partitioning, the process was repeated 100 times and the median values were reported.

Second, the cognitive and sMRI AD-PS scores for all the subjects were computed as described in sections 2.5–2.7. We evaluated the performance of the AD-PS scores across cognitive status groups and for groups of ADNI participants stratified according to age (<75 versus > = 75), and by the functional status. We used a cutoff value of 2 to stratify participants by their FAQ results. Discriminative maps for the different types of tissues were generated. In addition, to assess associations of all metrics with the distribution of times of MCI to AD conversion we performed survival analyses based on proportional hazards regression using SAS.

**Figure 1:**
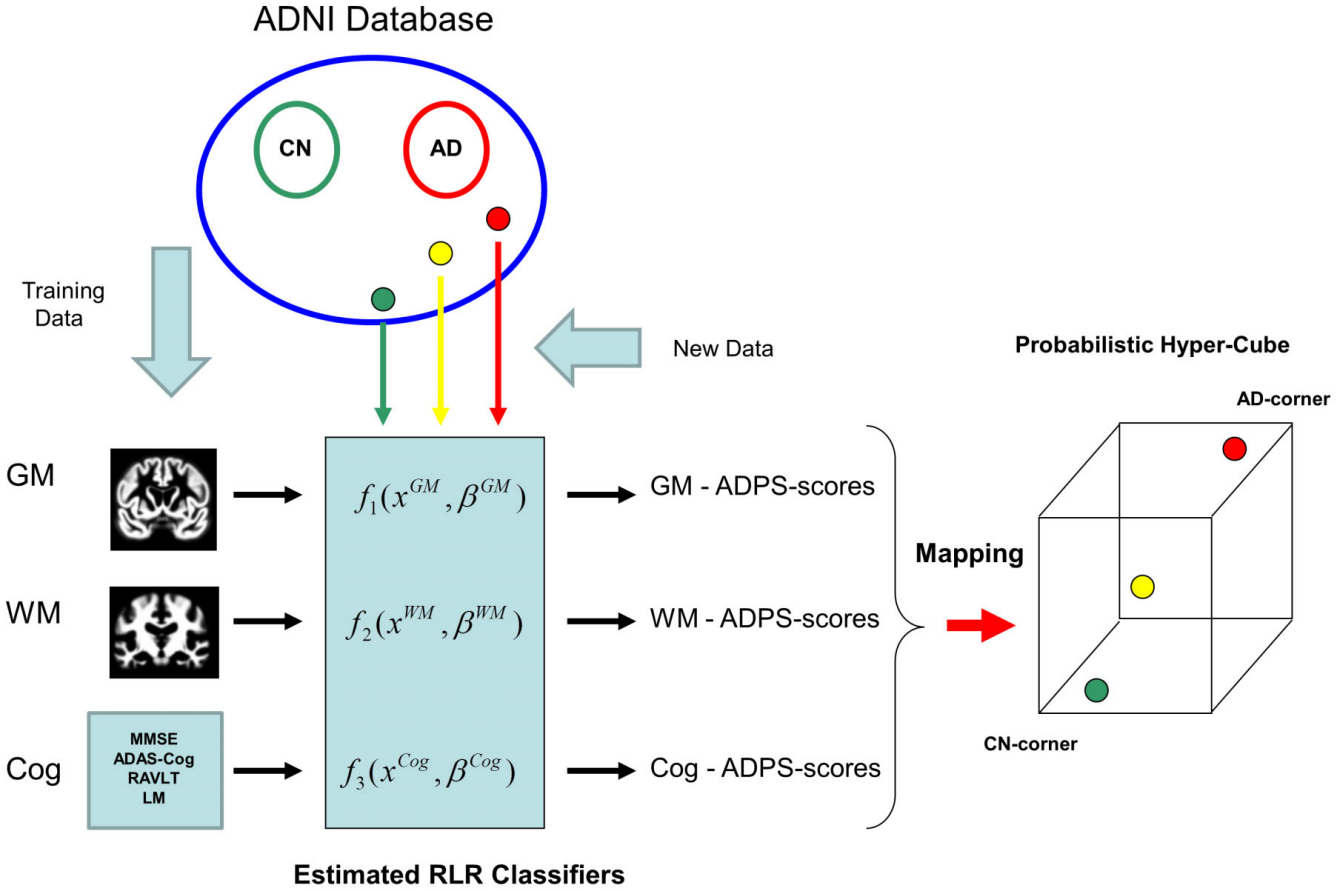
The concept of a probabilistic hypercube is illustrated. The probability hypercube can be interpreted as a geometrical representation of the output of a set of generative classifiers, each one estimated with different types of data. The set of AD-PS scores corresponding to a given individual define a position inside a unit hypercube. The position inside the hypercube for three individuals is illustrated.

Finally, in all analyses described above, we evaluated two composite metrics: 1) Anatomical AD-PS scores, which represent the sum of GM, WM, and CSF probabilities; and 2) Cognitive-anatomical AD-PS scores, which is the sum of the anatomical and cognitive scores. Since our scores are probabilities, their combinations can be interpreted as metrics defined in a unit multidimensional hypercube. In Figure 1, a scheme illustrating the probabilistic hypercube concept is presented. The probability hypercube can be interpreted as a geometrical representation of the output of a set of generative classifiers each one estimated with different types of data. Each type of information defines a dimension in the hypercube, and a set of AD-PS scores corresponding to one individual defines a position inside. Proximity to the corner (0,0,…0) is related to lower risk of AD, while proximity to other corners is associated with increased similarity between the patterns found in the given individual to those found in AD patients, thus signaling a greater risk. In particular, proximity of a given individual to the corner (1, 1,…., 1), which we call the “AD corner”, is associated with risk of AD across all sources of information. We provide from ADNI data two dimensional (GM versus WM) examples illustrating the concept of the probability hypercube. Statistical testing in the analyses described above was performed in all cases using the two sample Kolmogorov-Smirnov non-parametric test, which evaluates if the samples were drawn from the same distribution. SPARE-AD and total hippocampal volume performances were provided for comparison in all analyses described above.

## Results

**Figure 2:**
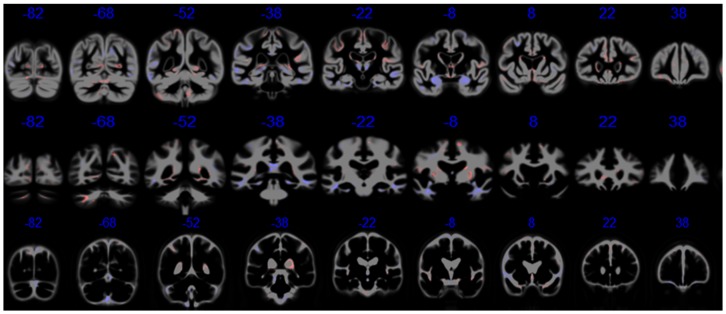
RLR classifier performances across different types of information and cognitive groups. Consistent with previous reports, grey matter (GM) tissue was more informative than white matter (WM) and cerebrospinal fluid (CSF). Interestingly, this difference decreases when a group with less severe cognitive decline is compared with the cognitively normal (CN) group.

**Figure 3:**
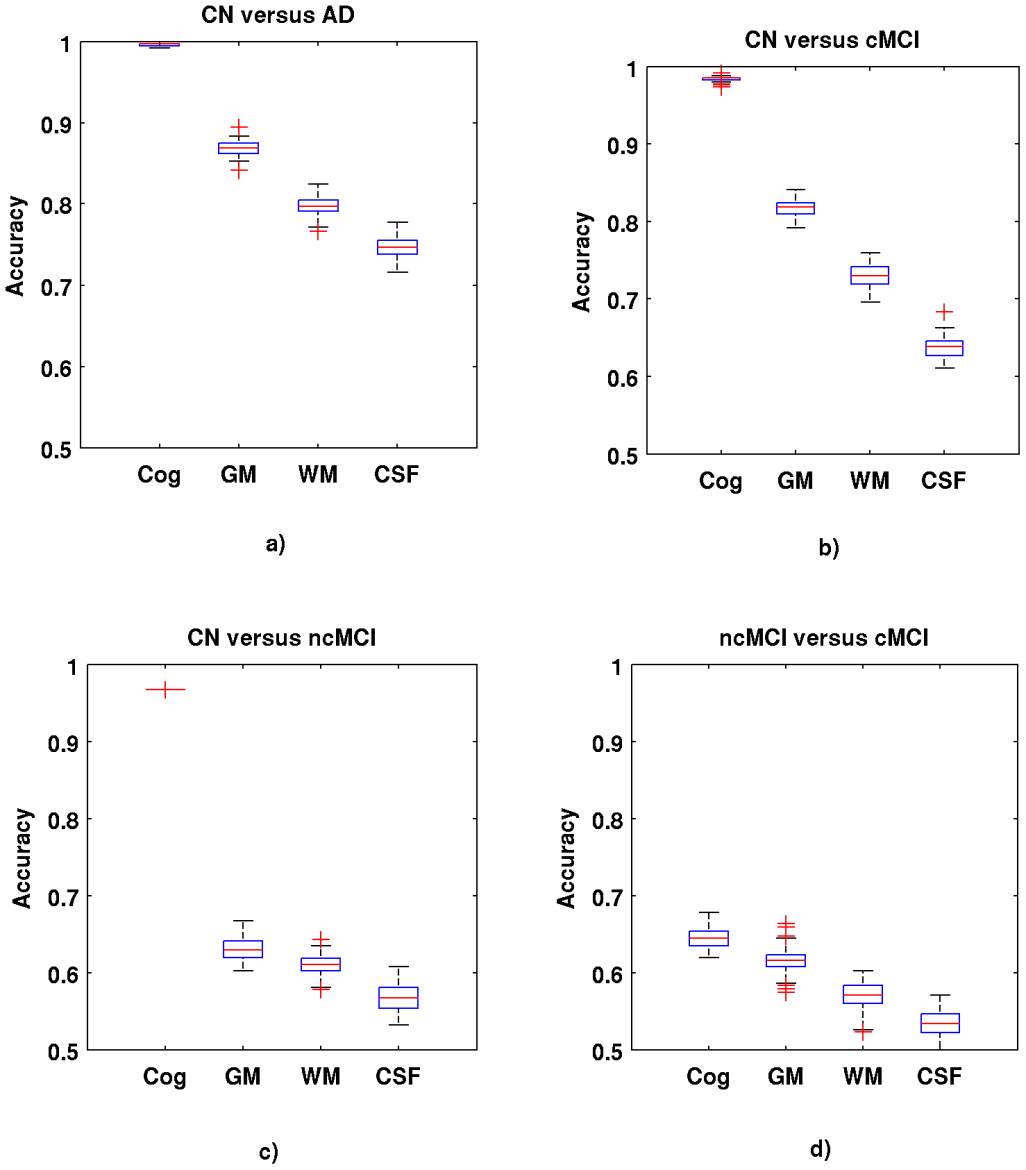
The GM, WM and CSF discriminative maps produced by logistic regression with sparsity regularization are overlaid on the study customized template generated by DARTEL. In each case, nine coronal slices (−82, −68, −52, −38, −22, −8, 8, 22, 38) are shown (neurological convention) in the first, second, and third rows, respectively. The blue areas are associated with AD classification, while the red ones are associated with CN classification.

Results of the first analyses are presented in Figure 2 and Table 3. The highest accuracy rates for classifying participants across all cognitive groups were achieved by cognitive classifiers. This was expected, since the cognitive data were used to assign participants into the clinical groups in advance which can be considered a type of overfitting. A different situation is discrimination of ncMCI and cMCI participants, since cognitive testing was not used to create these groups. They were determined by their change in classification over the 3 years of follow-up. Consistent with previous reports [31], [32] based on SVM methods, the GM was more informative than other types of brain tissues when discriminating CN versus AD. However, when discriminating CN from ncMCI, GM and WM tissue classifiers showed similar performance. In Table 3 we show median values of classification accuracy, sensitivity and specificity across cognitive groups and type of brain tissue. Results for classification based on cognitive testing are only shown for discrimination of ncMCI from cMCI participants for the reasons described above. In Figure 3 the GM, WM and CSF tissue discriminative maps produced by regularized logistic regression are presented in the first, second and third rows, respectively. The blue areas are those associated with AD classification, while the red ones are associated with CN classification. In the GM maps, we observed as relevant to classification several brain regions that have been widely associated with AD such as the hippocampus, parahippocampal gyrus, medial temporal lobe, thalamus and parietal lobe. The WM maps show areas in the temporal lobe adjacent to temporal lobe areas highlighted in the GM maps (e.g. parahippocampal gyrus), anterior and posterior corpus callosum while the CSF maps show clearly the ventricles.

**Table 3:** Median values of classification accuracy, sensitivity and specificity across cognitive groups are presented.

Classification Case	Information	Accuracy	Sensitivity	Specificity
	GM	**87.1**	**84.3**	**88.9**
CN versus AD	WM	79.6	76.8	82.6
	CSF	74.6	70.6	78.0
	GM	81.5	74.0	88.1
CN versus cMCI	WM	72.3	66.3	78.9
	CSF	63.9	50.4	74.2
	GM	63.0	58.6	68.1
CN versus ncMCI	WM	61.4	58.2	65.5
	CSF	56.8	49.9	64.2
	Cognitive	64.5	57.9	70.1
ncMCI versus cMCI	GM	61.5	45.8	75.5
	WM	57.4	35.7	76.9
	CSF	53.4	31.2	73.1

Results for cognitive data-based classifiers are only shown for cMCI versus ncMCI classification. In other situations, their performance is close to 100% because cognitive data have been used to generate the cognitive groups in advance.

The AD-PS scores significantly distinguished participants grouped according to clinical status (see Table 4). All AD-PS scores showed a clear increasing trend with poorer cognitive status. In Table 5 the results of the survival analyses are presented. The AD-PS composite cognitive-anatomical score was more strongly associated with MCI to AD conversion times and the machine learning generated scores (e.g. AD-PS and SPARE-AD) showed often stronger associations than the more conventional total hippocampal volume. In Tables 6–7, we present results of the estimation of the AD-PS scores for subjects stratified by age, and FAQ scores for each clinical group, respectively. The GM, WM and CSF scores often detected significantly greater AD-like patterns in participants over 75 years, while the cognitive scores did not reflect significant differences AD-like cognitive patterns. Finally, we observed significantly greater AD-like cognitive, GM and WM patterns in ncMCI with FAQ values above 2, while AD patients with FAQ values above 2 showed increased AD-like patterns in GM, WM and CSF tissues.

**Table 4:** Median AD-PS scores by type of information, SPARE-AD index and total hippocampal volume at baseline are presented.

scores	CN	ncMCI	p-value
AD-PS Cognitive	0.008	0.58	_5.6*10_^−54^
AD-PS GM	0.16	0.41	4.8*10^−11^
AD-PS WM	0.29	0.43	1.3*10^−10^
AD-PS CSF	0.36	0.39	9.0*10^−3^
AD-PS Anatomical	0.75	1.24	3.4*10^−11^
AD-PS Composite	0.79	1.83	6.7*10^−30^
SPARE-AD	−1.43	0.33	4.2*10^−52^
THV	7326	6523	2.4*10^−8^

scores	ncMCI	cMCI	p-value
AD-PS Cognitive	0.58	0.89	6.4*10^−8^
AD-PS GM	0.41	0.69	3.6*10^−10^
AD-PS WM	0.43	0.58	2.7*10^−6^
AD-PS CSF	0.38	0.56	_1.1*10_^−4^
AD-PS Anatomical	1.24	1.79	_2.9*10_^−8^
AD-PS Composite	1.83	2.54	_1.2*10_^−10^
SPARE-AD	0.33	1.11	_1.9*10_^−9^
THV	6523	5904	1.1*10^−6^

scores	cMCI	AD	p-value
AD-PS Cognitive	0.89	0.99	_1.8*10_^−28^
AD-PS GM	0.69	0.84	_1.1*10_^−3^
AD-PS WM	0.58	0.72	_2.9*10_^−3^
AD-PS CSF	0.56	0.66	2.0*10^−3^
AD-PS Anatomical	1.79	2.15	_4.1*10_^−5^
AD-PS Composite	2.54	3.14	_1.1*10_^−7^
SPARE-AD	1.11	1.35	_0.03_
THV	5904	5478	1.9*10^−2^

**Table 5:** Results from proportional hazards regression to assess associations with the distribution of times until conversion to AD.

Factor	Hazard Ratio Per 1 SD Unit in Score	95% Confidence Interval	z-statistic	P-value
AD-PS Cog scores	2.126	1.720, 2.627	6.981	1.47*10^−12^
AD-PS GM scores	1.803	1.512, 2.150	6.562	2.7*10^−11^
AD-PS WM scores	1.391	1.185, 1.633	4.028	2.8*10^−5^
AD-PS CSF scores	1.391	1.184, 1.635	4.005	3.1*10^−5^
Anatomical	1.643	1.395, 1.935	5.944	1.4*10^−9^
Composite	1.968	1.651, 2.346	7.554	2.1*10^−14^
SPARE-AD	1.763	1.480, 2.101	6.339	1.6*10^−10^
THV	1.597	1.344, 1.894	5.339	4.7*10^−8^

**Table 6:** Median values of AD-PS scores, SPARE-AD index and total hippocampal volume were estimated across cognitive status categories for ADNI participants, based on age (<75 yrs., vs. ≥75 yrs.).

	CN			ncMCI			cMCI			AD		
Age (#subjects)	<75 (86)	> = 75 (102)	p-value	<75 (102)	> = 75(80)	p-value	<75 (68)	> = 75 (85)	p-value	<75 (75)	> = 75 (96)	p-value
Cog	0.009	0.007	0.86	0.56	0.61	0.53	0.87	0.89	0.55	0.99	0.99	0.4
GM	0.09	0.22	0.0004	0.31	0.44	0.02	0.60	0.76	0.004	0.78	0.85	0.45
WM	0.20	0.31	0.0001	0.39	0.47	0.03	0.54	0.68	0.001	0.63	0.76	0.004
CSF	0.24	0.33	0.002	0.29	0.42	0.01	0.43	0.67	0.0008	0.61	0.73	0.009
Anatomical	0.57	0.91	4.5*10^−6^	1.1	1.4	0.004	1.5	2.1	7.6*10^−5^	2.1	2.2	0.07
Composite	0.60	0.93	6.7*10^−6^	1.7	1.9	0.003	2.3	2.9	1.5*10^−4^	3.1	3.20	0.08
SPARE-AD	−1.48	−1.31	0.1	0.05	0.57	2.4*10^−4^	0.88	1.37	1.6*10^−3^	1.22	1.39	0.034
THV	7674	7060	4.7*10^−4^	6858	6240	2.4*10^−4^	6226	5561	3.8*10^−4^	5684	5245	0.007

AD-PS Anat. stands for anatomical score (GM+WM+CSF).

**Table 7:** Median values of AD-PS scores, SPARE-AD index and total hippocampal volume were estimated across clinical groups by functional status (FAQ≤2 v. >2).

Metrics	ncMCI			cMCI			AD		
	= <2(70)	>2(112)	p-value	= <2(52)	>2(101)	p-value	= <2(10)	>2(161)	p-value
Cog	0.45	0.74	0.02	0.89	0.87	0.85	0.98	0.99	0.30
GM	0.36	0.46	0.03	0.69	0.68	0.73	0.56	0.84	0.016
WM	0.41	0.49	0.01	0.62	0.57	0.39	0.60	0.72	0.11
CSF	0.35	0.42	0.22	0.52	0.57	0.37	0.52	0.66	0.35
Anatomical	1.14	1.38	0.03	1.83	1.80	0.86	1.77	2.16	0.18
Composite	0.65	2.09	4.5*10^−3^	2.54	2.50	0.98	2.66	3.15	0.03
SPARE-AD	0.24	0.62	0.037	1.18	1.09	0.89	1.03	1.36	0.13
THV	6712	6281	0.052	5893	5928	0.63	6479	5337	2.4*10^−4^

Data for CN is not presented because all CN subjects except one had FAQ scores below 2. Numbers of subjects in each group are indicated in parentheses.

**Figure 4:**
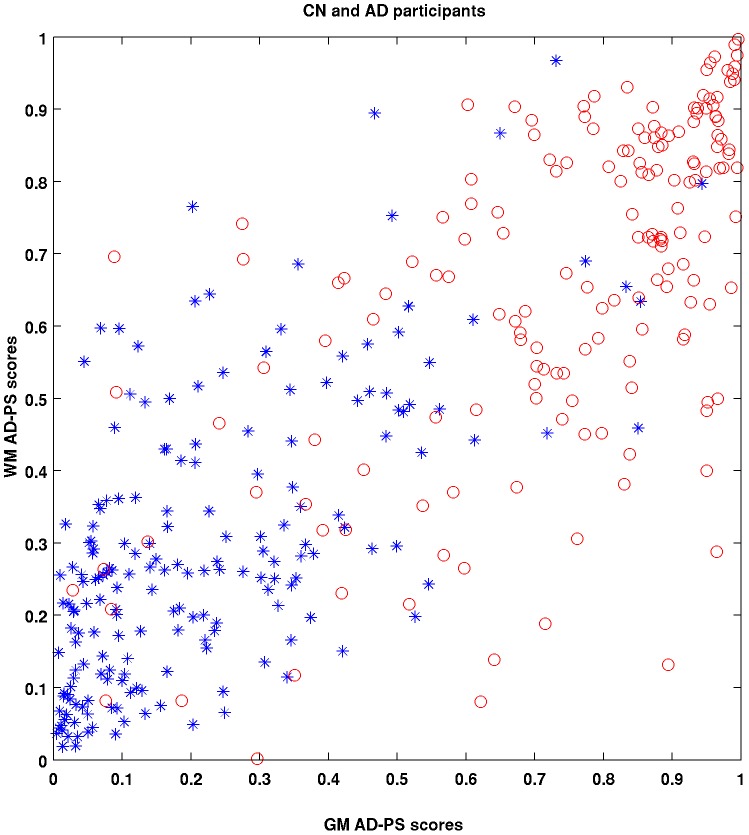
Two-dimensional probabilistic hypercube views of ADNI data showing AD-PS grey matter (GM) and white matter (WM) scores for 188 cognitively normal (CN – blue stars) and 171 Alzheimer's disease (AD) patients (red circles). They tended to cluster in different corners, as expected.

In Figure 4, the concept of the probability hypercube is illustrated using ADNI data. The spatial distribution inside a two dimensional GM versus WM hypercube of 188 CN and 171 AD ADNI participants according to their AD-PS scores is presented. CN and AD participants tended to cluster towards different corners in the plots. Most of the CN subjects (blue starts) were located closer to the (0, 0) corner, a zone of lower risk, while the AD patients (red circles) were closer to the corner (1, 1), an area of higher risk. Interestingly, some AD participants had low anatomical risk and some CN participants had high risk. Also, in a four dimensional (GM-WM-CSF-Cognitive) hypercube, we observed that 85% of the MCI subjects inside the area of lower risk (<0.5 for all types of data) remained stable, while 76% of the MCI participants inside the area of higher risk (>0.5 for all types of data) progressed to AD. However, the MCI subjects falling in these two areas represented only 37% of the total number of MCI subjects in our study.

## Discussion

The main goal of this analysis was to introduce and test new metrics for assessment of AD risk. Similar metrics, such as the SPARE-AD and STAND score [3], [5], have been proposed previously to detect AD-like abnormalities using structural MRI data. Both are based on the use of SVMs combined with severe dimension reduction measures. Alternatively, our AD-PS scores are based on the solution of classification problems of very large size via the use of logistic regression with sparsity regularization. We used the conditional probabilities modeled by large-scale regularized logistic regression as metrics, describing the similarity of the anatomical patterns found in a given individual to those found in AD patients. Despite the high dimensionality of the voxel space, our approach is relatively fast. Furthermore, due to the elastic net regularization, it produces voxel-based and sparse discriminative maps indicating the brain areas more relevant to prediction. In addition, we have extended the approach to cognitive data. The AD-PS cognitive scores are composite scores that detect AD-like anomalies based on cognitive scores taken from several memory tests in ADNI. Previously, composite cognitive scores to assess AD risk have been proposed by others [7], [8], but using a different rationale. While we used the conditional probabilities generated by regularized logistic regression, they used RF and psychometric theory methods to generate composite scores. Our scores, instead of providing a measure of the cognitive function in a more classical sense, are similarity measures of the cognitive patterns found in a given individual to those found in AD patients. The AD-PS cognitive scores often did not capture significant differences across age groups within a given cognitive status, which could be a consequence of the cognitive information being used to generate the cognitive groups. In Table 4, AD-PS cognitive scores tended to be close to zero and close to one for CN and AD participants, respectively. However, we still found significant correlations of the ncMCI, cMCI and AD participants' AD-PS cognitive scores with their corresponding THV and SPARE-AD scores (see Tables S1–S2 in File S1) and very strong associations with time of MCI to AD conversion. AD-PS cognitive scores also indicated significantly greater risk of AD in ncMCI participants with more impaired functional status. We expect that longitudinal follow-up may provide a better reflection of age-related cognitive change. The approach is not limited in any way to the 25 cognitive outcome measures we selected here to illustrate the concept. We will incorporate other cognitive tests (e.g. executive function) available through ADNI in the future.

We systematically evaluated the relevance of different types of brain tissue when discriminating cognitive status groups in the voxel space. If we take as a reference recent work in the ADNI literature [19], [33], our results for GM compare very well to those reported for SVM methods. Our approach did less well when discriminating ncMCI from cMCI, but other sMRI-based methods did not do much better in the studies noted above. In fact, classification methods based on the voxel space in [19] performed worse than our RLR approach, but the ROI-based ones did better in terms of sensitivity and specificity. This is a situation of great clinical importance that very likely requires the inclusion of other types of data (e.g. PET, amyloid biomarkers, etc.) for better discrimination, combined with dimension reduction such as using ROI data [34] and principal component analysis [35] or much larger sample sizes for methods based on voxel space like ours. The comparisons we made to previous work must be interpreted with caution, because CV procedures and sample sizes were different from ours. Meaningful statements about relative performance only can be made when the methods are tested under the same conditions, as done by Cuingnet and colleagues [19]. Tables 8–9 contain information about relative performance of different methods in the literature together with details about sample size, CV technique and normalization method. These tables highlight the great variety of conditions based on which metrics of classifier performance were estimated. Table 10 reports results related to detection of differences between ncMCI and cMCI participants of the ADNI dataset using machine learning generated metrics and statistical testing. The results reported by Hinrichs and colleagues differ in terms of types of data and statistical testing, sample sizes, and time of conversion from MCI to AD, which makes their results difficult to compare directly to ours.

**Table 8:** Summary of classification reports of CN versus AD subjects based on structural MRI data from different groups.

MLM	Info	SS	NM	CV Method	Acc	Sens	Spec
RLR – Casanova et al.	GM -voxels	359 (188 CN) -ADNI	DARTEL	Ten folds with three-ways-split – 100 repetitions	87.1	84.3	88.9
Linear SVM Chu et al. 2012	GM-voxels	262 (131CN) - ADNI	DARTEL	Leave one out	84.3	NA	NA
Linear SVM –Cuignet.et al. 2011	GM- voxels	182 (81 CN) -ADNI	DARTEL	Two fold (137 for testing) with fixed partitioning	NA	81	95
Linear SVM - Zhang et al. 2011	93 anatomical ROIs	103 (52 CN) - ADNI	HAMMER	Ten folds with three-ways-split – 10 repetitions	86.2	86.0	86.3
Vemuri et al. 2008	GM+WM+CSFVoxels*	280 (140 CN) –Mayo clinic	SPM5 methods	Two fold (100 for testing) with fixed partitioning	NA	86.0	86.0
COMPARE - Fan et al. 2008.	GM+WM+CSF	122 (66 CN) - ADNI	HAMMER	Leave one out	94%	NA	NA
COMPARE - Cuignet.et al. 2011	GM	182 (81 CN) -ADNI	DARTEL	Two fold (137 for testing) with fixed partitioning	NA	82.0	89.0
Logistic Regression –Teipel et al. 2007	GM+WM - PCA	50 (18 CN)	SPM2 methods	None	83.0	88.0	78.0

**MLM = Machine Learning Method; SS** = Sample size; **NM** = Normalization Method; **CV** = Cross-validation,

* -Images were downsampled.

**Table 9:** Summary of classification reports of ncMCI versus cMCI subjects based on structural MRI data from different groups.

MLM	Info	SS	NM	CV Method	Acc	Sens	Spec
RLR – Casanova et al.	GM -voxels	335 (182 cMCI) -ADNI	DARTEL	Ten folds with three-ways-split – 100 repetitions	63.0	58.6	68.1
Linear SVM Chu et al. 2012	Voxels in ROI	180 (90 ncMCI) - ADNI	DARTEL	Leave one out	65.0	NA	NA
Linear SVM Chu et al. 2012	GM-voxels	180 (90 ncMCI) - ADNI	DARTEL	Leave one out	58.0	NA	NA
Linear SVM –Cuignet.et al. 2011	GM- voxels	106 (39 cMCI) -ADNI	DARTEL	Two fold (104 for testing) with fixed partitioning	NA	0	100
COMPARE - Misra et al. 2009.	GM+WM+CSF	103 (76 ncMCI) - ADNI	HAMMER	Leave one out	81.5	NA	NA
COMPARE - Cuignet.et al. 2011	GM	106 (39 cMCI) -ADNI	DARTEL	Two fold (137 for testing) with fixed partitioning	NA	62.0	67.0
Logistic Regression –Teipel et al. 2007	CSF - PCA	24 (9 ncMCI)	SPM2 methods	None	80	67	93

**MLM = Machine Learning Method; SS** = Sample size; **NM** = Normalization Method; **CV** = Cross-validation.

**Table 10:** Results related to detection of differences between ncMCI from cMCI ADNI participants based on statistical testing and imaging data.

Scores	Info	SS	p-values
AD-PS – Casanova et al.	GM –voxels (ADNI)	335 (182 ncMCI) -ADNI	3.6*10^−10^
AD-PS – Casanova et al.	Anatomical (ADNI)	335 (182 ncMCI) -ADNI	2.9*10^−8^
SPARE-AD –Casanova et al.	GM+WM+CSF (ADNI)	335 (182 ncMCI) -ADNI	1.9*10^−9^
*MMDM – Hinrichs et al. 2011	MRI+PET (ADNI)	119 MCI – ADNI	1.8*10^−6^

Hinrichs et al. compared means using a t-test instead of distributions as we have done in this work.

* MCI to AD conversion was within a year of follow-up.

Our approach generates discriminative maps at a voxel level which uncover brain regions that have been associated with AD before (e.g. hippocampus, parahippocampal gyrus, etc.). They are similar in interpretation to those generated by a linear SVM [18] but while those are dense, ours due to the elastic net regularization are sparse pinpointing brain regions relevant to classification. Exploratory voxel-wise analyses (not presented) showed that the blue areas correspond to brain regions with significant decreases of tissue volume (tissue atrophy). The interpretation of the red areas is more subtle. Two sample SPM t-tests in GM produced significant results mostly in areas located in the boundary of GM and CSF, which are known to be challenging for segmentation algorithms [36]. Vemuri et al. 2008 suggested that the presence of these areas is the result of noise in the data due to partial volume effects, segmentation and registration errors, etc. This issue requires further study.

There is a growing body of literature indicating increasing interest in the role of white matter in AD [37]–[41]. Several studies have identified volume loss in various portions of the corpus callosum as related to AD [42]. The callosal white matter loss has been related to Wallerian degeneration, receiving axons from the temporo-parietal regions involved in AD. In the field of imaging genetics, interest in WM is also growing. Several groups are beginning to report associations of apolipoprotein E and other genetic markers with WM tissue integrity and atrophy [43]–[45]. However, often machine learning studies in the literature have focused on the role of GM, whole brain or ROI, and the roles of WM and CSF have been less investigated. In our study, similar to previous reports, we found GM to be more discriminative when classifying CN versus AD subjects. However, relative performance of WM with respect to GM increased when the CN group was compared to a group with less severe cognitive status than AD. When discriminating MCI subjects with stable cognitive status from CN subjects, GM and WM classifiers' performance is similar. This suggests that WM could play a more important role in early stages of AD than previously thought. Interestingly, for CN participants the WM AD-PS scores were greater than their corresponding GM counterparts (see Table 4), a trend that still can be observed in ncMCI participants but not in cMCI or AD participants, whose AD-like anomalies are much greater in GM than in WM. Similar observations were made for CN subjects across age groups. WM AD-PS scores showed more significant differences between age groups than the corresponding GM AD-PS scores (see Table 6). Interestingly, our discriminative maps show adjacency of WM and GM patterns of atrophy, especially in the temporal lobe, a brain region believed to be affected early by AD. Thus, although AD has been traditionally thought to be predominantly a disease of GM tissue, our findings support previous reports that suggest a role of the WM in early stages of the disease [46], [47].

We proposed here metrics for AD risk assessments which integrate information from different sources by combining probabilities generated by classifiers. Similar ideas have been used previously in face and voice recognition based on Bayesian theory [48]. The probability hypercube concept that we have introduced can be interpreted as a geometrical representation of the output of a set of generative classifiers, each one estimated with different types of data. It is intuitive, it provides a natural environment to generate multimodal metrics for AD detection, and it can be a powerful paradigm to visualize information in a clinical AD database such as ADNI. Two- or three-dimensional graphics of the AD-PS scores can offer researchers and clinicians a quick intuitive understanding of how the participants are located according to given biomarkers indicating AD-like abnormalities, and also to locate groups of participants whose assigned cognitive status does not correspond to the estimated risk.

Here the AD-PS scores were estimated using cognitive and structural data independently. We generated combinations of the scores to seek a composite cognitive-anatomical metric for AD risk assessment with increased performance compared with metrics based on a specific source of information. While many different composite metrics could be devised, in this work we used the sum of the scores across sources of information to illustrate the concept. In some situations, our composite metrics improved detection of differences of distributions between clinical groups, such as ncMCI from cMCI participants, but very often it did not. This could suggest that collapsing all the multimodal information in a single score may not always be useful and/or the non-optimality of the composite metric used here.

The AD-PS and SPARE-AD scores very often detected more significant differences between groups than THV. Although in general the AD-PS scores often produced more significant results, in several situations they were outperformed by the SPARE-AD scores and THV. These relative results do not represent a rigorous comparison of these three metrics. For example, the AD-PS and SPARE-AD scores are estimated using different image processing approaches, sample sizes and smoothing kernels. On the other hand, the THV used here were based on FreeSurfer estimates; there are other estimators available that were not tested here that could be more accurate. The SPARE-AD and THV performances were instead provided as a reference to help assess and validate the AD-PS scores. Additional information about correlations of the three metrics across cognitive groups can be found in Tables S1 and S2 in File S1.

Our study has several limitations. A potential confounding factor here is the quality of the brain tissue segmentation. Although we made an effort to generate masks covering each type of tissue, there could be overlap among areas. We centered our analyses on the use of only sMRI and cognitive data because these were available for most ADNI-1 participants at baseline. In the future, we will estimate the AD-PS scores for amyloid PET imaging, amyloid and tau levels in CSF, etc. Our composite cognitive scores included only a portion of the cognitive data available in ADNI: memory scores. We chose these parameters because of their well-documented association with AD and their use in previous work by other researchers. We will include additional cognitive information in the future. High performance of cognitive data-based classifiers is in large part the result of these data being used to define cognitive groups in advance, which gives these classifiers an unfair advantage. Our sMRI AD-PS scores were based on images normalized using DARTEL; although this is a method easy to use and less time-consuming than other methods in the field, it may not be the best option. We expect significant improvements of AD-PS anatomical scores by using more sophisticated normalization methods. It is very likely the AD-PS scores will benefit from increasing the sample size, which could be implemented by integrating the ADNI databases to be available worldwide in the coming years [1]. The sum composite metric chosen here assigns similar weights to different modalities, which is very likely non-optimal. We will evaluate in the future different metrics defined within the probability hypercube. Also, we did not adjust for multiple comparisons in our analyses, but we often observed the expected trends in the values of the scores across clinical severity and groups of participants ordered by higher risk. To evaluate performance of the scores, we used the Kolmogorov-Smirnov two-sample test, which is only one of several possible choices. Censored ncMCI cognitive data were only considered in the survival analyses. If some of those censored ncMCI participants converted to AD within 36 months, other ncMCI versus cMCI discrimination results are very likely slightly worse than they should be. Finally, one of the regularization parameters was fixed empirically to avoid additional computations. A finer selection could lead to further improvements of the results presented here.

## Conclusion

Our analyses provided evidence of the validity of the AD-PS scores. In general the AD-PS scores distinguished well between AD-like cognitive and anatomical patterns across clinical status, as seen in the gradient of values across clinical groups ordered by severity. The structural AD-PS scores often detected greater AD-like abnormalities in older and less functional ADNI participants according to the FAQ. The differences in AD-like patterns detected by the AD-PS scores were always in the expected directions across cognitive status, age and functional groups. In addition, they also were consistent with directions detected by other known metrics such as SPARE-AD and THV. The survival analyses showed that the AD-PS scores are strongly associated to the MCI to AD conversion times. The AD-PS metrics can be a powerful tool in AD research to detect AD-like cognitive and anatomical effects across given groups of subjects stratified by clinical, risk factors or intervention groups. Finally, the approaches presented here can be extended to other neurodegenerative diseases such as Parkinson's, amyotrophic lateral sclerosis, etc. This will be the subject of future work.

## Supporting Information

File S1 **File S1 contains supplementary materials. Figure S1.** The CV procedure with nested 10 fold CV is illustrated. The RLR model is estimated for all different values of the grid (alpha is fixed in our case) using the internal training data. The values of the regularization parameters that produced maximum accuracy when tested on the internal testing dataset are recorded. The process is repeated 10 times using different internal folds as testing dataset. At the end the average value of the recorded regularization parameters is computed and the RLR model is recomputed using the external training data set. The external testing dataset is used to estimate classification accuracy, sensitivity and specificity which are recorded. The above process is repeated ten times across the ten external folds and the final estimator of the three metrics is computed as their average across the ten external folds. **Table S1.** Correlations (p-values) between AD-PS and SPARE-AD scores across cognitive statuses computed using the Spearman's rank sum test are presented. **Table S2.** Correlations (p-values) between AD-PS and SPARE-AD scores and THV across cognitive statuses computed using the Spearman's rank sum test are presented. **Table S3.** The IDs of the 188 CN participants are listed. **Table S4.** The IDs of the 171 participants are listed. **Table S5.** The IDs of the 153 MCI converters participants are listed. **Table S6.** The IDs of the 182 MCI non-converters participants are listed.
(DOCX)
