# Supplementary material for: Alzheimer's Disease Risk Assessment Using Large-Scale Machine Learning Methods
Source: PLoS One. 2013 Nov 8;8(11):e77949. doi: 10.1371/journal.pone.0077949 (PMC3826736; doi:10.1371/journal.pone.0077949)
Supplement: File S1 — contains supplementary materials. Figure S1. The CV procedure with nested 10 fold CV is illustrated. The RLR model is estimated for all different values of the grid (alpha is fixed in our case) using the internal training data. The values of the regularization parameters that produced maximum accuracy when tested on the internal testing dataset are recorded. The process is repeated 10 times using different internal folds as testing dataset. At the end the average value of the recorded regularization parameters is computed and the RLR model is recomputed using the external training data set. The external testing dataset is used to estimate classification accuracy, sensitivity and specificity which are recorded. The above process is repeated ten times across the ten external folds and the final estimator of the three metrics is computed as their average across the ten external folds. Table S1. Correlations (p-values) between AD-PS and SPARE-AD scores across cognitive statuses computed using the Spearman's rank sum test are presented. Table S2. Correlations (p-values) between AD-PS and SPARE-AD scores and THV across cognitive statuses computed using the Spearman's rank sum test are presented. Table S3. The IDs of the 188 CN participants are listed. Table S4. The IDs of the 171 participants are listed. Table S5. The IDs of the 153 MCI converters participants are listed. Table S6. The IDs of the 182 MCI non-converters participants are listed. (DOCX) [file pone.0077949.s001.docx]

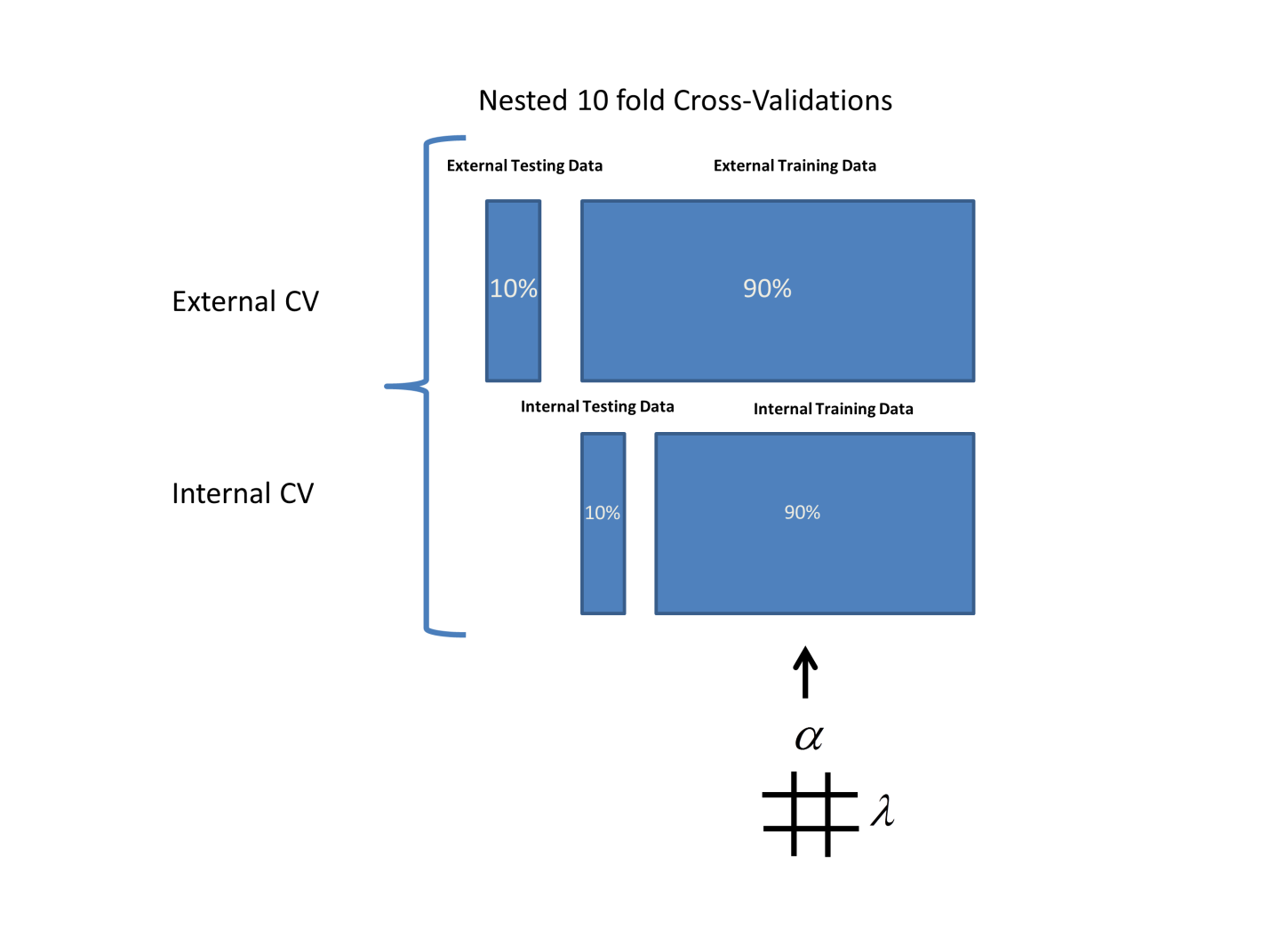


Fig S1 – The CV procedure with nested 10 fold CV is illustrated. The RLR model is estimated for all different values of the grid (alpha is fixed in our case) using the internal training data. The values of the regularization parameters that produced maximum accuracy when tested on the internal testing dataset are recorded. The process is repeated 10 times using different internal folds as testing dataset. At the end the average value of the recorded regularization parameters is computed and the RLR model is recomputed using the external training data set. The external testing dataset is used to estimate classification accuracy, sensitivity and specificity which are recorded. The above process is repeated ten times across the ten external folds and the final estimator of the three metrics is computed as their average across the ten external folds.

Table S1 – Correlations (p-values) between AD-PS and SPARE-AD scores across cognitive statuses computed using the Spearman’s rank sum test are presented.

|  | **Cognitive** | **GM** | **WM** | **CSF** | **Anatomical** |
| --- | --- | --- | --- | --- | --- |
| **CN** | -0.11 (0.12) | 0.35 (1.2*10^-6^) | 0.32 (1.2*10^-6^) | 0.06 (0.41) | 0.31 (1.8*10^-5^) |
| **ncMCI** | 0.41 (1.3*10^-8^) | 0.75 (0) | 0.61 (0) | 0.46 (0.9*10^-12^) | 0.76 (0) |
| **cMCI** | 0.23 (0.005) | 0.66 (0) | 0.57 (0) | 0.43 ( 0.3*10^-9^) | 0.67 (0) |
| **AD** | 0.15 (0.045) | 0.36 (2.0*10^-6^) | 0.23 (0.003) | 0.23 (0.003) | 0.45 (1.6*10^-5^) |

Table S2 – Correlations (p-values) between AD-PS and SPARE-AD scores and THV across cognitive statuses computed using the Spearman’s rank sum test are presented.

|  | **Cognitive** | **GM** | **WM** | **CSF** | **Anatomical** | **SPARE-AD** |
| --- | --- | --- | --- | --- | --- | --- |
| **CN** | 0.14 (0.051) | -0.52 (1.0*10^-14^) | -0.41 (6.8*10^-9^) | -0.41 (5.6*10^-9^) | -0.56 (1.0*10^-16^) | -0.21 (0.001) |
| **ncMCI** | -0.33 (7.1*10^-6^) | -0.64 (2.1*10^-22^) | -0.55 (1.6*10^-15^) | -0.39 (5.8*10^-8^) | -0.64 (1.8*10^-22^) | -0.65 (1.5*10^-23^) |
| **cMCI** | -0.20 (0.01) | -0.47 (0.9*10^-11^) | -0.47 (1.4*10^-9^) | -0.33 (2.3*10^-5^) | -0.50 (3.2*10^-11^) | -0.58 (6.4*10^-15^) |
| **AD** | -0.42 (0.9*10^-10^) | -0.38 (4.2*10^-7^) | -0.37 (1.0*10^-8^) | -0.18 (0.016) | -0.37 (4.9*10^-7^) | -0.26 (4.4*10^-4^) |

Table S3 – The IDs of the 188 CN participants are listed.

|  | Cognitive Normal | Participants |  |
| --- | --- | --- | --- |
| ID | ID | ID | ID |
| 002_S_0295 | 021_S_0159 | 051_S_1123 | 116_S_1232 |
| 002_S_0413 | 021_S_0337 | 052_S_0951 | 116_S_1249 |
| 002_S_0559 | 021_S_0647 | 052_S_1251 | 123_S_0072 |
| 002_S_0685 | 021_S_0984 | 057_S_0643 | 123_S_0106 |
| 002_S_1261 | 022_S_0066 | 057_S_0818 | 123_S_0113 |
| 003_S_0907 | 022_S_0096 | 057_S_0934 | 123_S_0298 |
| 003_S_0931 | 022_S_0130 | 062_S_0578 | 126_S_0405 |
| 003_S_0981 | 023_S_0031 | 062_S_0768 | 126_S_0506 |
| 003_S_1021 | 023_S_0058 | 067_S_0019 | 126_S_0605 |
| 005_S_0553 | 023_S_0081 | 067_S_0177 | 126_S_0680 |
| 005_S_0602 | 023_S_0926 | 067_S_0257 | 127_S_0259 |
| 005_S_0610 | 023_S_0963 | 068_S_0127 | 127_S_0260 |
| 006_S_0484 | 023_S_1190 | 068_S_0473 | 127_S_0622 |
| 006_S_0498 | 023_S_1306 | 068_S_1191 | 128_S_0229 |
| 006_S_0681 | 024_S_0985 | 072_S_0315 | 128_S_0245 |
| 006_S_0731 | 027_S_0074 | 073_S_0089 | 128_S_0272 |
| 007_S_0068 | 027_S_0118 | 073_S_0311 | 128_S_0500 |
| 007_S_0070 | 027_S_0120 | 073_S_0312 | 128_S_0522 |
| 007_S_1206 | 027_S_0403 | 073_S_0386 | 128_S_0545 |
| 009_S_0751 | 029_S_0824 | 082_S_0304 | 128_S_0863 |
| 009_S_0842 | 029_S_0845 | 082_S_0363 | 129_S_0778 |
| 009_S_0862 | 029_S_0866 | 082_S_0640 | 130_S_0886 |
| 010_S_0067 | 031_S_0618 | 082_S_0761 | 130_S_0969 |
| 010_S_0419 | 032_S_0095 | 082_S_1256 | 131_S_0123 |
| 010_S_0420 | 032_S_0479 | 094_S_0489 | 131_S_0319 |
| 010_S_0472 | 032_S_0677 | 094_S_0692 | 131_S_0436 |
| 011_S_0002 | 032_S_1169 | 094_S_0711 | 131_S_0441 |
| 011_S_0005 | 033_S_0516 | 094_S_1267 | 131_S_1301 |
| 011_S_0008 | 033_S_0734 | 098_S_0171 | 133_S_0433 |
| 012_S_0637 | 033_S_0741 | 098_S_0172 | 133_S_0488 |
| 012_S_1133 | 033_S_0920 | 098_S_0896 | 133_S_0493 |
| 012_S_1212 | 033_S_0923 | 099_S_0040 | 133_S_0525 |
| 013_S_0502 | 033_S_1016 | 099_S_0090 | 136_S_0086 |
| 013_S_0575 | 033_S_1086 | 099_S_0352 | 136_S_0184 |
| 013_S_1035 | 033_S_1098 | 099_S_0533 | 136_S_0186 |
| 013_S_1276 | 035_S_0156 | 099_S_0534 | 136_S_0196 |
| 014_S_0519 | 035_S_0555 | 100_S_0015 | 137_S_0283 |
| 014_S_0520 | 036_S_0576 | 100_S_0035 | 137_S_0301 |
| 014_S_0558 | 036_S_0672 | 100_S_0047 | 137_S_0459 |
| 016_S_0359 | 036_S_0813 | 100_S_0069 | 137_S_0686 |
| 016_S_0538 | 036_S_1023 | 100_S_1286 | 137_S_0972 |
| 018_S_0043 | 037_S_0303 | 114_S_0416 | 141_S_0717 |
| 018_S_0055 | 037_S_0327 | 114_S_0601 | 141_S_0726 |
| 018_S_0369 | 037_S_0454 | 116_S_0360 | 141_S_0767 |
| 018_S_0425 | 041_S_0125 | 116_S_0382 | 141_S_0810 |
| 020_S_0097 | 041_S_0262 | 116_S_0648 | 141_S_1094 |
| 020_S_1288 | 041_S_1002 | 116_S_0657 | 941_S_1195 |

Table S4 – The IDs of the 171 participants are listed.

|  | AD Participants |  |  |
| --- | --- | --- | --- |
| IDs | IDs | IDs | IDs |
| 002_S_0619 | 021_S_0753 | 051_S_1296 | 126_S_0606 |
| 002_S_0816 | 021_S_1109 | 053_S_1044 | 126_S_0784 |
| 002_S_0938 | 022_S_0129 | 057_S_0474 | 126_S_0891 |
| 002_S_1018 | 022_S_0543 | 057_S_1371 | 126_S_1221 |
| 003_S_1059 | 023_S_0083 | 057_S_1373 | 127_S_0431 |
| 003_S_1257 | 023_S_0084 | 057_S_1379 | 127_S_0754 |
| 005_S_0221 | 023_S_0093 | 062_S_0535 | 127_S_0844 |
| 005_S_0814 | 023_S_0139 | 062_S_0690 | 127_S_1382 |
| 005_S_0929 | 023_S_0916 | 062_S_0730 | 128_S_0167 |
| 005_S_1341 | 023_S_1262 | 062_S_0793 | 128_S_0216 |
| 006_S_0547 | 023_S_1289 | 067_S_0029 | 128_S_0266 |
| 006_S_0653 | 024_S_1171 | 067_S_0076 | 128_S_0310 |
| 007_S_0316 | 024_S_1307 | 067_S_0110 | 128_S_0517 |
| 007_S_1248 | 027_S_0404 | 067_S_0812 | 128_S_0528 |
| 007_S_1304 | 027_S_0850 | 067_S_1185 | 128_S_0740 |
| 007_S_1339 | 027_S_1081 | 067_S_1253 | 128_S_1409 |
| 009_S_1334 | 027_S_1082 | 068_S_0109 | 128_S_1430 |
| 010_S_0786 | 027_S_1254 | 073_S_0565 | 130_S_1290 |
| 010_S_0829 | 029_S_0836 | 082_S_1079 | 130_S_1337 |
| 011_S_0003 | 029_S_0999 | 082_S_1377 | 131_S_0457 |
| 011_S_0010 | 029_S_1056 | 094_S_1027 | 131_S_0497 |
| 011_S_0053 | 029_S_1184 | 094_S_1090 | 131_S_0691 |
| 011_S_0183 | 031_S_0321 | 094_S_1102 | 133_S_1055 |
| 012_S_0689 | 031_S_1209 | 094_S_1164 | 133_S_1170 |
| 012_S_0712 | 032_S_0147 | 094_S_1397 | 136_S_0194 |
| 012_S_0720 | 032_S_0400 | 094_S_1402 | 136_S_0299 |
| 012_S_0803 | 032_S_1037 | 098_S_0149 | 136_S_0300 |
| 013_S_0592 | 032_S_1101 | 098_S_0884 | 136_S_0426 |
| 013_S_0699 | 033_S_0724 | 099_S_0372 | 137_S_0366 |
| 013_S_0996 | 033_S_0733 | 099_S_0470 | 137_S_0438 |
| 013_S_1161 | 033_S_0739 | 099_S_0492 | 137_S_0796 |
| 013_S_1205 | 033_S_0889 | 099_S_1144 | 137_S_0841 |
| 014_S_0328 | 033_S_1281 | 100_S_0743 | 137_S_1041 |
| 014_S_0356 | 033_S_1283 | 100_S_0747 | 141_S_0790 |
| 014_S_1095 | 033_S_1285 | 100_S_1062 | 141_S_0852 |
| 016_S_0991 | 033_S_1308 | 100_S_1113 | 141_S_0853 |
| 016_S_1263 | 035_S_0341 | 114_S_0979 | 141_S_1024 |
| 018_S_0286 | 036_S_0577 | 116_S_0392 | 141_S_1137 |
| 018_S_0335 | 036_S_0759 | 116_S_0487 | 141_S_1152 |
| 018_S_0633 | 036_S_0760 | 116_S_1083 |  |
| 018_S_0682 | 037_S_0627 | 123_S_0088 |  |
| 020_S_0213 | 041_S_1368 | 123_S_0091 |  |
| 021_S_0343 | 041_S_1391 | 123_S_0094 |  |
| 021_S_0642 | 041_S_1435 | 123_S_0162 |  |

Table S5 - The IDs of the 153 MCI converters participants are listed.

|  | MCI converters | |  |
| --- | --- | --- | --- |
| ID | ID | ID | ID |
| 002_S_0729 | 023_S_0217 | 037_S_0566 | 116_S_0834 |
| 002_S_0954 | 023_S_0331 | 037_S_1225 | 116_S_1243 |
| 002_S_1070 | 023_S_0388 | 041_S_0314 | 116_S_1271 |
| 005_S_0222 | 023_S_0604 | 041_S_0549 | 116_S_1315 |
| 005_S_0572 | 023_S_0625 | 041_S_1412 | 123_S_0050 |
| 005_S_1224 | 023_S_0855 | 041_S_1423 | 126_S_0708 |
| 006_S_0675 | 023_S_0887 | 041_S_1425 | 126_S_0865 |
| 006_S_1130 | 023_S_1247 | 051_S_1331 | 126_S_1077 |
| 007_S_0041 | 024_S_1393 | 052_S_0952 | 127_S_1427 |
| 007_S_0101 | 027_S_0179 | 052_S_1054 | 128_S_0227 |
| 007_S_0128 | 027_S_0256 | 053_S_0389 | 128_S_0258 |
| 007_S_0249 | 027_S_0408 | 053_S_0507 | 128_S_0611 |
| 007_S_0344 | 027_S_0461 | 057_S_0839 | 128_S_0947 |
| 010_S_0904 | 027_S_0835 | 057_S_0941 | 128_S_1148 |
| 011_S_0326 | 027_S_1213 | 057_S_1007 | 130_S_0289 |
| 011_S_0856 | 027_S_1387 | 057_S_1217 | 130_S_0423 |
| 011_S_0861 | 029_S_1073 | 057_S_1265 | 131_S_1389 |
| 011_S_1282 | 031_S_0294 | 062_S_1299 | 132_S_0987 |
| 012_S_1033 | 031_S_0568 | 067_S_0045 | 133_S_0638 |
| 012_S_1292 | 031_S_1066 | 067_S_0098 | 133_S_0727 |
| 013_S_0240 | 032_S_0187 | 067_S_0243 | 133_S_0913 |
| 013_S_0325 | 032_S_0214 | 067_S_0336 | 136_S_0195 |
| 013_S_0860 | 032_S_0978 | 068_S_0442 | 136_S_0429 |
| 014_S_0563 | 033_S_0511 | 068_S_0476 | 136_S_0695 |
| 014_S_0658 | 033_S_0513 | 073_S_0518 | 136_S_0873 |
| 016_S_0702 | 033_S_0514 | 094_S_0434 | 136_S_0874 |
| 016_S_1326 | 033_S_0567 | 094_S_1015 | 137_S_0631 |
| 018_S_0057 | 033_S_0723 | 094_S_1398 | 137_S_0973 |
| 018_S_0080 | 033_S_0725 | 098_S_0269 | 141_S_0697 |
| 018_S_0155 | 033_S_0906 | 098_S_0667 | 141_S_0915 |
| 018_S_0450 | 033_S_0922 | 099_S_0054 | 141_S_0982 |
| 021_S_0141 | 033_S_1116 | 099_S_0111 | 141_S_1004 |
| 021_S_0231 | 035_S_0204 | 100_S_0190 | 141_S_1244 |
| 022_S_0750 | 035_S_0997 | 100_S_0892 | 941_S_1295 |
| 022_S_1394 | 036_S_0869 | 100_S_0930 | 941_S_1311 |
| 023_S_0030 | 036_S_0976 | 100_S_0995 | 941_S_1363 |
| 023_S_0042 | 036_S_1135 | 100_S_1154 |  |
| 023_S_0078 | 036_S_1240 | 116_S_0649 |  |
| 023_S_0126 | 037_S_0182 | 116_S_0752 |  |

Table S6 - The IDs of the 182 MCI non-converters participants are listed.

|  | non-converter MCI Participants | | |
| --- | --- | --- | --- |
| ID | ID | ID | ID |
| 002_S_0782 | 027_S_0307 | 052_S_0989 | 116_S_0361 |
| 002_S_1155 | 027_S_0417 | 052_S_1346 | 116_S_0890 |
| 002_S_1268 | 027_S_0485 | 053_S_0621 | 126_S_0709 |
| 003_S_0908 | 027_S_0644 | 053_S_0919 | 126_S_1187 |
| 003_S_1074 | 027_S_1045 | 057_S_0464 | 126_S_1340 |
| 005_S_0324 | 027_S_1277 | 057_S_0957 | 127_S_0397 |
| 005_S_0448 | 029_S_0914 | 057_S_1269 | 127_S_0925 |
| 005_S_0546 | 029_S_1038 | 062_S_1182 | 127_S_1032 |
| 007_S_0293 | 029_S_1215 | 062_S_1294 | 127_S_1210 |
| 007_S_0414 | 029_S_1218 | 067_S_0038 | 127_S_1419 |
| 007_S_0698 | 029_S_1318 | 067_S_0176 | 128_S_0135 |
| 009_S_1030 | 029_S_1384 | 067_S_0284 | 128_S_0138 |
| 011_S_1080 | 031_S_0351 | 067_S_0290 | 128_S_0225 |
| 012_S_0634 | 031_S_0821 | 067_S_0607 | 128_S_0715 |
| 012_S_0932 | 031_S_0830 | 068_S_0401 | 128_S_0770 |
| 012_S_1165 | 031_S_0867 | 068_S_0802 | 128_S_1406 |
| 012_S_1175 | 032_S_0718 | 068_S_0872 | 129_S_1204 |
| 012_S_1321 | 033_S_1279 | 068_S_1075 | 129_S_1246 |
| 013_S_1120 | 033_S_1284 | 072_S_1211 | 130_S_0102 |
| 013_S_1186 | 033_S_1309 | 072_S_1380 | 130_S_0285 |
| 013_S_1275 | 035_S_0033 | 073_S_0445 | 130_S_0449 |
| 014_S_0169 | 036_S_0656 | 073_S_0746 | 130_S_0505 |
| 014_S_0557 | 036_S_0673 | 073_S_1357 | 130_S_0783 |
| 016_S_0354 | 036_S_0748 | 082_S_0469 | 131_S_0409 |
| 016_S_0590 | 036_S_0945 | 082_S_0641 | 132_S_0339 |
| 016_S_1149 | 037_S_0150 | 082_S_0832 | 133_S_0629 |
| 018_S_0087 | 037_S_0377 | 082_S_0928 | 133_S_0771 |
| 018_S_0103 | 037_S_0501 | 082_S_1119 | 133_S_0792 |
| 018_S_0142 | 037_S_0552 | 094_S_0531 | 133_S_1031 |
| 021_S_0178 | 037_S_0588 | 094_S_0921 | 136_S_0107 |
| 021_S_0273 | 037_S_1078 | 094_S_1293 | 136_S_0579 |
| 021_S_0276 | 037_S_1421 | 094_S_1314 | 136_S_1227 |
| 021_S_0332 | 041_S_0407 | 094_S_1330 | 137_S_0158 |
| 021_S_0424 | 041_S_0446 | 094_S_1417 | 137_S_0481 |
| 021_S_0626 | 041_S_0598 | 098_S_0160 | 137_S_0800 |
| 022_S_0044 | 041_S_0679 | 099_S_0051 | 137_S_0825 |
| 022_S_0544 | 041_S_0721 | 099_S_0060 | 137_S_0994 |
| 022_S_0924 | 041_S_1260 | 099_S_0291 | 137_S_1414 |
| 022_S_0961 | 041_S_1411 | 099_S_0880 | 137_S_1426 |
| 022_S_1097 | 041_S_1418 | 099_S_1034 | 141_S_0851 |
| 022_S_1351 | 041_S_1420 | 100_S_0006 | 141_S_1051 |
| 023_S_0376 | 051_S_1040 | 100_S_0296 | 141_S_1052 |
| 023_S_0613 | 051_S_1072 | 114_S_0410 | 141_S_1231 |
| 023_S_1046 | 051_S_1131 | 114_S_1103 | 141_S_1255 |
| 024_S_1400 | 051_S_1338 | 114_S_1106 |  |
| 027_S_0116 | 052_S_0671 | 114_S_1118 |  |
